# Core measures for developmentally supportive care in neonatal intensive care units: theory, precedence and practice

**DOI:** 10.1111/j.1365-2648.2009.05052.x

**Published:** 2009-10

**Authors:** Mary Coughlin, Sharyn Gibbins, Steven Hoath

**Affiliations:** Mary Coughlin MS RN Global Clinical Services Manager Children’s Medical Ventures NorwellMassachusetts, USA; Sharyn Gibbins PhD RN Head of Interdisciplinary Research & Evidence Based Practice Sunnybrook Women’s Hospital TorontoOntario, Canada; Steven Hoath MD Medical Director, Skin Science Institute Professor of Pediatrics, Division of Neonatology Cincinnati Children’s Hospital Medical CenterOhio, USA

**Keywords:** core measures, developmentally supportive care, neonatal intensive care unit, nursing

## Abstract

**Title:**

Core measures for developmentally supportive care in neonatal intensive care units: theory, precedence and practice.

**Aim:**

This paper is a discussion of evidence-based core measures for developmental care in neonatal intensive care units.

**Background:**

Inconsistent definition, application and evaluation of developmental care have resulted in criticism of its scientific merit. The key concept guiding data organization in this paper is the United States of America’s Joint Commission’s concept of ‘core measures’ for evaluating and accrediting healthcare organizations. This concept is applied to five disease- and procedure-independent measures based on the Universe of Developmental Care model.

**Data sources:**

Electronically accessible, peer reviewed studies on developmental care published in English were culled for data supporting the selected objective core measures between 1978 and 2008. The quality of evidence was based on a structured predetermined format that included three independent reviewers. Systematic reviews and randomized control trials were considered the strongest level of evidence. When unavailable, cohort, case control, consensus statements and qualitative methods were considered the strongest level of evidence for a particular clinical issue.

**Discussion:**

Five core measure sets for evidence-based developmental care were evaluated: (1) protected sleep, (2) pain and stress assessment and management, (3) developmental activities of daily living, (4) family-centred care, and (5) the healing environment. These five categories reflect recurring themes that emerged from the literature review regarding developmentally supportive care and quality caring practices in neonatal populations. This practice model provides clear metrics for nursing actions having an impact on the hospital experience of infant-family dyads.

**Conclusion:**

Standardized disease-independent core measures for developmental care establish minimum evidence-based practice expectations and offer an objective basis for cross-institutional comparison of developmental care programmes.

**What is already known about this topic**

- Developmental care has been a recognized practice strategy in neonatal intensive care units for over two decades.
- Developmental care has been linked to a variety of favourable clinical outcomes.
- There is inconsistency in the definition and operationalization of developmental care.

**What this paper adds**

- Five core measure sets for evidence-based developmental care were identified: protected sleep, pain and stress assessment and management, developmental activities of daily living, family-centred care and the healing environment.
- These five categories reflect recurring themes that emerged from the literature review regarding developmentally supportive care and quality caring practices in neonatal populations.
- This practice model provides clear metrics for nursing actions having an impact on the hospital experience of infant-family dyads.

**Implications for practice and/or policy**

- Application of evidence-based ‘core measures’ drawn from the field of developmental care and used for disease-independent evaluation of neonatal nursing care should demonstrate clinical, psychoemotional and economic benefits which can be quantified as outcomes.
- The core measures concept for developmental care should standardize care experiences for patients, families and staff during neonatal intensive care unit stays.
- Core measures for developmental care should validate and quantify the impact of nursing care activities in neonatal intensive care units, allowing cross-institutional comparisons.

## Introduction

Developmental care for high-risk infants in neonatal intensive care units (NICUs) is practised throughout the industrialized world. Developmental care is a professional practice, education and research opportunity that nurses need to explore, evaluate and refine continuously within the rapidly changing technological environment of the NICU. Although the practice and philosophical interpretation of developmental care may vary across units, the goal is to provide a structured care environment which supports, encourages and guides the developmental organization of the premature and/or critically ill infant. Developmental care recognizes the physical, psychological and emotional vulnerabilities of premature and/or critically ill infants and their families and is focused on minimizing potential short and long-term complications associated with the hospital experience.

Developmental care has its roots in the principles of nursing science as outlined by Florence Nightingale (1860) indicating the nurses responsibility in creating and maintaining an environment conducive to the healing process. These principles, in conjunction with the early work of pioneer neonatal nurses and paediatricians, laid the theoretical foundation for the work of Als and colleagues (Als 1982, Als *et al.* 1988a, 1988b), who described the complex relationship between the developing brain of preterm infants and the increasingly technological NICU environment.

Based on the premise that infant behaviours are a means of communication, healthcare professionals were encouraged to examine infant responses to the environment systematically and adjust their caregiving activities when signs of stress were observed. Subsequent researchers have tested the general hypothesis that the provision of a developmentally appropriate sensory milieu, coupled with minimal disruptions and care contingent on patient cues, improves medical and developmental outcomes.

In two systematic reviews and one meta-analysis, developmental care has been shown to decrease length of hospital stay and hospital costs and improve weight gain and time to full enteral feeding, as well as to improve neurodevelopmental scores at 9–12 months (Jacobs *et al.* 2002, Symington & Pinelli 2002,Symington & Pinelli 2006). Despite these documented benefits, we concluded that confusion about the existing theoretical construct and the inability to identify and measure relevant clinical outcomes reliably has resulted in inconsistent adoption of developmental care and undermined its potential as a revolutionary and transformative healthcare philosophy and practice paradigm.

## Background

**Figure 1:**
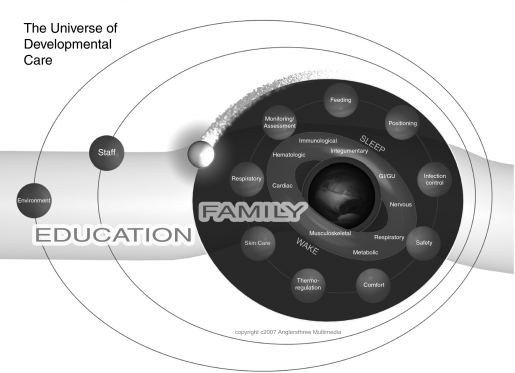
The Universe of Development Care.

In an attempt to extend Als’ Synactive Theory as a theoretical foundation for developmental care, the Universe of Developmental Care model (UDC; Gibbins *et al.* 2008) was proposed to create a practical heuristic framework highlighting developmental care practices in a patient- and family-centric context. The model focused the caregiver’s attention to the interface linking the body/organism and the environment, specifically referred to as the *shared care surface* (Figure 1). The concept of a shared care surface was advanced as a logical cornerstone for neonatal nursing care. This surface was conceptualized as the place where body and environment meet. It is the context as well as the actual location where care occurs. Neither the developing infant nor the environment exist in isolation, but rather intersect at this shared surface. In care interactions, there are not two separate surfaces bumping against each other or two separate surfaces with an intervening ‘space’, but a single, continuous, looped structure which is both organism and environment. Thus, observations about comfort, safety, tolerance, health, wellness and satisfaction within this complex dynamic system of the patient care experience can be made relative to this confluence.

### Conceptual model

The UDC model portrays a patient-centric care environment, graphically representing the patient at the center of the healthcare universe (Figure 1). The patient is illustrated as a dynamic organism consisting of internal physiological systems influenced by a requisite sleep-wake cycle and an outer care surface (the planetary ring). It is a disturbance within the physiological orbit which necessitates medical intervention and nursing care. Access to these internal body systems occurs across the shared care surface. The shared care surface is the observable boundary of the infant where care takes place.

The family is placed intentionally as proximal as possible to the infant-patient. This placement acknowledges the crucial role of family in the patient’s hospital experience and creates a visual reminder of this relationship to the clinician. The staff is depicted in a protective orbit around the infant-family dyad. Beyond the staff is the environment, comprised of the physical, human and organizational elements that represent the healthcare setting.

The universe as a whole is situated within an educational medium which pervades and connects individual constituents. In this model, education extends across all orbital planes. Family, staff and healthcare organizations have unique learning needs that cannot be ignored within a healthcare milieu that is dedicated to quality healthcare delivery and outcomes. The UDC model is an extension to existing nursing knowledge and is proposed as a means to critically examine individual or collective components of developmental care in order to evaluate practice, identify research questions and/or identify learning opportunities related to care practices. Translation of developmental care into practice requires language and definitions that clearly articulate expectations through measurable, objective and evidence-based criteria.

### Core measures

Focused attention on quality healthcare delivery is relatively new, beginning in the mid-1980s. At the turn of the 21st century, two landmark reports drew global attention to the quality healthcare crisis in the United States of America –*To Err is Human* (1999) and *Crossing the Quality Chasm* (2001). Around the globe, industrialized countries began to scrutinize the quality of their healthcare delivery and identify deficiencies and opportunities for improvement. In 1999, the Quality of Healthcare in America Committee stated that it was unacceptable for patients to be harmed by a healthcare system that was supposed to offer healing and comfort – a system that promised ‘First, do no harm’ (NAS 1999).

In 1999, the US Joint Commission, a not-for-profit organization that accredits and certifies healthcare organizations to ensure the safety and quality of patient care, collaborated with various international healthcare stakeholders to identify opportunities to improve disease management and reduce mortality. Despite the availability of effective evidence-based medical interventions for common life-threatening medical conditions, there was a high degree of variability in use of these proven therapies in the patient care setting. With a focus on quality care delivery through the application of standardized medical treatment strategies, evidence-based medical interventions were organized into disease-specific core measure sets across several life-threatening medical conditions. Each core measure set is comprised of attributes which target, define, and specify the scientifically valid and reliably applied actions needed to achieve improvement. Corresponding, measurable criteria articulate the specific actions needed to achieve the designated attribute.

Through the definition of clear, measurable benchmarks for clinical practice, the Joint Commission’s core measure concept has reduced mortality in the area of heart failure, acute myocardial infarction and community acquired pneumonia (Jha *et al.* 2007). Although these core measures have improved patient and system outcomes within disease-specific areas (Jha *et al.* 2005), processes to standardize complex care practices, such as those involved in developmental care, have not been explored.

### Core measures for developmental care

**Figure 2:**
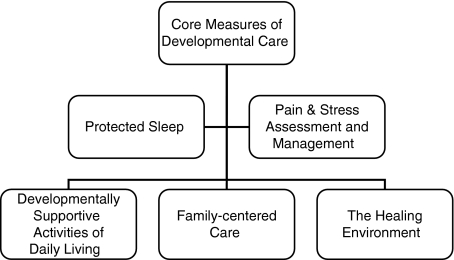
The core measures of developmental care.

Unlike the criteria employed by the Joint Commission to manage discrete medical conditions, core measures for developmental care are focused on care actions which are disease-independent but nonetheless essential to promote healthy growth and development of the infant and family. The proposed five core measures represent the first step in operationalizing evidence-based developmental care (Figure 2). The core measures are protected sleep, pain and stress assessment and management, activities of daily living (positioning, feeding and skin care), family-centred care and the healing environment. These five categories reflect the recurring themes that emerged from the literature review regarding developmentally supportive care and quality caring practices in the neonatal population. Each core measure set represents an organized constellation of caring activities that acknowledges the holistic needs of the infant-family dyad within the context of the UDC model and the hospital experience. Presenting care strategies in this format creates a reflective opportunity for care providers, taking the focus off the care ‘task’ and placing it on the care ‘experience’ at the shared care surface.

## Data sources

To define and standardize developmental care in the context of the UDC and Joint Commission’s core measures concept, a comprehensive electronic search was conducted in MEDLINE, CINAHL, EMBASE and PsycINFO using the terms ‘developmental care’, ‘developmentally supportive care’, ‘caring’, and ‘infant’ between 1978 and 2008. Papers were selected for inclusion if they identified specific interventions within the five core measures that improved short or long-term morbidity outcomes.

Evaluating the quality of evidence was based on a predetermined structured format and involved three independent reviewers. Systematic reviews and randomized control trials were considered the strongest level of evidence. When not available, cohort, case control, consensus statements and qualitative methods were considered the strongest level of evidence for a particular phenomenon of interest.

## Discussion

The patient-centeredness of the UDC model aligns seamlessly with the Joint Commission core measures concept. The UDC approach seeks to frame evidence-based, developmentally supportive care practices and integrate these practices into a performance measurement system similar to the Joint Commission core measures model. The strategic identification and implementation of core measures can then provide consistency in the interpretation and clinical application of developmental care.

### Core measure 1: protected sleep

Protected sleep is the most important core measure because it highlights the importance of behavioural state; which is the foundation for all human activities. Only when an individual is physically, behaviourally and emotionally prepared for interaction can caregiving activities occur without deleterious effects (Periano & Algarin 2007,Fitzgerald *et al.* 1998). The attributes pertaining to protected sleep encompass assessment, documentation and utilization of infant state to guide care delivery (Holditch-Davis *et al.* 2003,Grigg-Damberger *et al.* 2007). The corresponding criteria include specific interventions that promote sleep (Feldman *et al.* 2002, Schmidt 2004, Ludington-Hoe *et al.* 2006), and educate families about the importance of sleep in the hospital as well as post-discharge at home (Task Force on Sudden Infant Death Syndrome 2005, Ludington-Hoe *et al.* 2006) (Table 1).

**Table 1:** Protected sleep core measure

Attribute	Criteria
Infant sleep-wake states will be assessed, documented, and guides all infant interactions (Holditch-Davis *et al.* 2003, Grigg-Damberger *et al.* 2007)	1.All non-emergent caregiving is provided during wakeful states
	2.Sleep-wake states are assessed and documented
	3.Scheduled caregiving is contingent on the infant’s sleep-wake states and adapted accordingly
Care strategies that support sleep are individualized for each infant and documented (Feldman *et al.* 2002, Schmidt 2004, Ludington-Hoe *et al.* 2006, White 2007)	1.Caregiving activities that promote sleep (i.e. facilitative tuck, swaddled bathing and skin-to-skin care) are integrated into the patient’s daily care plan
	2.All caregiving activities are modified according to the infant’s state
	3.Light and sound levels are maintained within the recommended range; implement cycled lighting to support nocturnal sleep
Families are educated on the importance of sleep safety in the hospital and the home; this education is documented (Task Force on Sudden Infant Death Syndrome 2005, Ludington-Hoe *et al.* 2006)	1.Family education on caregiving activities that promote safe sleep is provided
	2.Parenting opportunities are provided to promote infant sleep
	3.Staff role model ‘Back to Sleep’ practices for families once the infant has demonstrated physiologic flexion of the upper body in supine

### Core measure 2: pain and stress assessment and management

Attributes and corresponding criteria specific to pain and/or stress assessment and management are: (1) routine assessment and documentation of pain and stress with an established pain/stress tool (Stevens & Gibbins 2002, Anand *et al.* 2006), (2) management of pain and stress before, during, and following all painful procedures with subsequent documentation of interventions and a return of the infant’s pain scores to pre-procedural baseline (Anand *et al.* 2006, Sharek *et al.* 2006), and (3) involvement in and sharing of a pain and stress management care plan with parents (Franck *et al.* 2001, 2004) (Table 2).

**Table 2:** Assessment & management of stress and pain core measure

Attribute	Criteria
Assessments of pain and/or stress are performed routinely and documented (Stevens & Gibbins 2002, Anand *et al.* 2006)	1.Each infant is assessed for pain and/or stress at a minimum every 4 hours or with each infant interaction
	2.Each infant is assessed for pain and/or stress during all procedures and caregiving activities
	3.A valid pain assessment tool is utilized
Pain and/or stress is managed before, during and after all procedures until the infant reaches their baseline; interventions and infant responses are documented (Stevens & Gibbins 2002, Anand *et al.* 2006, Sharek *et al.* 2006)	1.Non-pharmacologic and/or pharmacologic measures are utilized prior to all stressful and/or painful procedures
	2.Caregiving activities are adapted to minimize pain and stress
	3.Infant response to pain and/or stress relieving interventions is documented
Family is involved and informed of the pain and stress management plan of care for their infant(s); involvement and information sharing is documented (Franck *et al.* 2001, 2004, Nibert & Ondrejka 2005)	1.Parents are involved and informed of the pain and stress management plan of care for their hospitalized infant(s)
	2.Family education regarding infant pain and stress cues is provided
	3.Family is encouraged to provide comfort to their infant

### Core measure 3: developmental activities of daily living: positioning, feeding and skin care

The attributes and criteria for positioning includes a commitment to ensure proper postural support throughout the infant’s hospital stay, documentation and role modelling of appropriate positioning practices to parents and colleagues (Sweeney & Gutierrez 2002, Vaivre-Douret *et al.* 2004, Chizawsky & Scott-Findlay 2005). Distinct attributes and criteria for feeding focus on the appropriate use of non-nutritive sucking, employing infant feeding cues as a measure of infant feeding readiness and parental education and support of breastfeeding and the use of breastmilk (McCain 2003, Pinelli & Symington 2005, Ludwig & Waitzman 2007). Finally, attributes and corresponding criteria specific to skin care highlight the importance of accurate assessment and documentation of skin integrity and practices which protect the vulnerable skin surface (Lund *et al.* 2001) (Table 3).

**Table 3:** Developmentally supportive activities of daily living core measure

Attribute	Criteria
Positioning: Infant positioning is documented to provide comfort, safety, physiologic stability and support optimal neuromotor development (Sweeney & Gutierrez 2002, Vaivre-Douret *et al.* 2004, Chizawsky & Scott-Findlay 2005)	1.Each infant is positioned and handled in flexion, containment and alignment during all caregiving activities
	2.Infant position is evaluated with every infant interaction and modified to support symmetric development
	3.Positioning aides are gradually removed and Back to Sleep and Tummy to Play practices are implemented as the infant demonstrates physiologic flexion of the upper body in supine
Feeding: Feeding will be infant-driven, individualized, nurturing, functional and developmentally appropriate to ensure safety (McCain 2003, Pinelli & Symington 2005, Ludwig & Waitzman 2007)	1.Non-nutritive sucking is offered with each non-oral feeding contingent on the infant’s state
	2.Assessment of feeding readiness cues and the quality of the oral feeding is documented with each oral feeding encounter
	3.Education regarding the benefits of breastmilk is provided and family choice is supported
Skin-care: Infant skin integrity is assessed, protected and care is documented (Lund *et al.* 2001, Curley *et al.* 2003)	1.Infants are bathed no more frequently than every 3 days
	2.Skin integrity is assessed using a reliable assessment tool at least once per shift and documented. (Braden Q Scale or similar tool)
	3.The skin surface is protected during application, utilization and removal of adhesive products

### Core measure 4: family-centred care

The family-centred care core measure incorporates the tenets of the Institute for Family-centred Care and recognizes that families must have (1) unrestricted access to their infant (Johnson *et al.* 2004, Nibert & Ondrejka 2005), (2) assessment of their emotional and physical well-being and their evolving competence and confidence in parenting their infant (Doucette & Pinelli 2004, Kaaresen *et al.* 2006), and (3) access to resources and supports that assist them in their short and long term parenting needs (Doucette & Pinelli 2004) (Table 4).

**Table 4:** Family-centred care core measure

Attribute	Criteria
The family (defined by the infant’s parents and/or guardians) has 24-hour unrestricted access to their infant and is provided the opportunity to parent; family definition and participation is documented (Johnson *et al.* 2004, Nibert & Ondrejka 2005)	1.Family is offered the opportunity to be present and/or participate in medical rounds and change of shift report
	2.Family is offered the opportunity to be present during invasive procedures and/or resuscitative interventions
	3.Family is supported in parenting activities to include skin-to-skin care, holding, feeding activities, dressing, bathing, diapering, singing and all infant care interactions
The family’s level of emotional well-being and parental confidence and competence is assessed and documented weekly (Doucette & Pinelli 2004, Kaaresen *et al.* 2006)	1.Mental health professionals resource families weekly
	2.Family observations and input regarding their infant are sought by the clinical care providers and documented in the patient’s health records
	3.Health care providers share unbiased infant information weekly with the family
The family has access to resources and supports that assist in short term and long term parenting, decision making and parental well-being (Doucette & Pinelli 2004)	1.Families are invited to participate in a neonatal intensive care unit family support group
	2.Culturally sensitive family education on infant safety and infant care is available in various formats
	3.Resources for the social, spiritual and financial needs of families are provided

### Core measure 5: the healing environment

The attributes specific to the healing environment encompass the physical, human and organizational elements requisite for a safe and healing hospital experience. The criteria include the measurement and maintenance of recommended light and sound levels and assurance of physical and auditory privacy for families (Johnson *et al.* 2004, White 2007), promotion of effective communication, collaboration, and caring behaviours among the healthcare team (Brown *et al.* 2003, Ohlinger *et al.* 2003, Schmidt 2004), and documentation of evidence-based policies, procedures and resources to sustain the healing environment over time (Lafferty 2004, Schmidt 2004) (Table 5).

**Table 5:** Core measure for the healing environment

Attribute	Criteria
A quiet, dimly lit, private environment that promotes safety and sleep (Johnson *et al.* 2004, White 2007)	1.Continuous background sound and transient sound in the neonatal intensive care unit shall not exceed an hourly continuous noise level (Leq) of 45 decibels (dB) and an hourly L10 (the noise level exceeded for 10% of the time) of 50 dB. Transient sounds or Lmax (the single highest sound level) shall not exceed 65 dB
	2.Ambient light levels ranging between 10–600 lux and 1–60 foot candles shall be adjustable and measured at each infant bed space
	3.Physical and auditory privacy is afforded at each patient bed space
A collaborative healthcare team that emanates teamwork, mindfulness and caring (Brown *et al.* 2003, Ohlinger *et al.* 2003, Schmidt 2004)	1.Interdisciplinary care rounds occur at least weekly
	2.Direct care providers demonstrate caring behaviors which include adherence to hand hygiene protocols, cultural sensitivity, open listening skills and a sensitive relationship orientation
	3.Nurse-physician collaboration is defined, practiced, and reinforced on a daily basis
Evidence-based policies, procedures and resources are available to sustain the healing environment over time (Lafferty 2004, Schmidt 2004)	1.Core measures of developmental care provide the standard of care for all patient care providers
	2.Resources to support the implementation of developmental care as defined by the core measures are always available
	3.A system for staff accountability in the practice of developmental care as outlined by the core measures is operational

## Conclusion

The core measures for developmental care create a framework for the comparative analysis of developmental care practices and associated clinical outcomes across multiple healthcare systems. Core measures quantify otherwise invisible nursing actions in NICUs with measurable and tangible constructs that are essential for improvement and standardization. Nurses can use the UDC and its core measures to guide and evaluate clinical practice. Once it is determined that the infant is in a state of optimal readiness to engage in a caring exchange, as measured by their sleep-wake cycle and ability to sustain a mutual relationship, the developmentally supportive care provider (parent/nurse) can begin their caring exchange with the infant-patient. For example, while engaging in a developmentally supportive diaper change, consideration of light and sound levels within the immediate care area, positioning and comfort needs during the procedure, assessment of skin integrity and responses to handling, and whether or not the family wishes to participate in care is incorporated into this seemingly simple task.

Nurses can also use core measures as a framework for clarifying and enriching parental and staff knowledge of developmental care. Didactic teaching sessions or interactive learning opportunities in which caregivers experience the contrast of standard (such as experiencing loud noises, frequent handling or bright lights) vs. developmental care practices (as defined by the core measures) may be used to increase understanding of developmental care.

The nursing profession has a long history of creating and maintaining an environment conducive to the healing process. Nurse clinicians, educators and scholars are increasingly committed to advancing the science of developmental care in relation to nursing practice. The concepts of core measures provide a template for testing and evaluating the practice of developmental care in the clinical setting. Similar to existing adult studies examining the safety, efficacy and cost effectiveness of core measures (Kfoury *et al.* 2008, Groce 2007, Shabot 2005, Braun *et al.* 1999) developmental care core measures need to be further developed and explored. The UDC model should be a useful, physiologically grounded, flexible and logical framework to accomplish this goal.

Although tremendous advances in neonatal care and the developmental support offered to high risk infants have been made over the past three decades, variability in practice remains a constant concern that precludes systematic comparisons. The UDC model extends a logical and visual model of care to a practical set of core measures important for neonatal nursing practice. These measures may or may not relate to specific diseases, but they always link the patient as an individualized person to the specific surface (body/environment) where care is rendered and care is received. Clearly defined, measurable evidence-based clinical practice criteria (as outlined by the core measures) provide an objective reference point for developmental care practice improvement in the NICU. Nursing, medicine, and other healthcare professionals are invited to embrace this practical framework, integrate these care actions into their professional practice and evaluate clinical, economic and psychosocial outcomes as a consequence of this standardized model for developmental care.
